# Medical Opinion and Movement

**Published:** 1910-08-27

**Authors:** 


### Medical Opinion and Movement.

### Transplantation of Bone.

An interesting case of autoplasty by transplantation of a bone is recorded by Roosing in the *Hospitalstidende*. The patient, aged twenty-six, was afflicted with an osteosarcoma of the upper third of the humerus. By a semicircular incision around the acromion process the shoulder joint was exposed. A second incision was carried from the first half-way down the external aspect of the arm. The upper end and shaft of the humerus were isolated, but the muscular insertions and periosteum were left adherent. The long biceps tendon was divided and the humerus was divided at the junction of the middle and lower thirds. The wound was plugged and the right fibula was rapidly isolated and the upper portion resected and removed, so that it was three centimetres longer than the portion of humerus resected. The cut end of the bone was pointed so as to fit into the medullary canal of the humerus, and the head of the fibula was fitted into the glenoid cavity. The capsule was sutured, and the muscles were sutured to the fibromuscular tags specially left on the surface of the fibula. The wound was then closed with a small drainage at the upper end. The wound healed well, and two months after operation the movements of the limb were quite free, and sufficient for feeding, brushing the hair, etc.; only abduction was limited. Radiography showed the fibula in position without any atrophy. The use of the leg was quite normal.

### Abdominal Auscultation.

In cases of large abdominal tumours it is often of considerable importance to determine before operating the presence of adhesions to the adjacent parts. According to Dr. V. Piazza-Martini, of Palermo, auscultation over the tumour affords frequently valuable information in this respect. If the mass is adherent the heart-sounds are clearly heard through the abdominal wall in the adherent zone, and often the respiratory murmur can be detected at the same time, and even the voice-sounds are transmitted. In three cases reported by this author these observations were confirmed by subsequent laparotomy. One was that of a large ovarian cyst. The heart-sounds were transmitted as far as the pubis and the groins, and the respiratory murmur could also be heard feebly. Operation showed a cyst with a twisted pedicle and adherent in every direction. The other two cases were also ovarian cysts, and the signs were similarly confirmed by laparotomy. In two other cases of ovarian cyst in which there were no adhesions these signs were also absent.

### Anaphylaxis.

Since the phenomena of anaphylaxis have been clearly defined and demonstrated a good deal of further research has been expended on the question, especially with a view to prevent its occurrence following therapeutic injections of serum in the human individual. According to Drs. Carnot and Slavu, the condition may be avoided by adding to the serum to be injected a certain quantity of hydrochloric acid. By experiments with guinea-pigs that have been previously sensitised, they find that the addition of 33 per cent. is sufficient to inhibit the production of anaphylactic symptoms, and this amount is too weak to interfere with the antitoxic properties of the serum. On the other hand, Dr. Besedka has worked out what appears to be a simpler solution of the problem. He finds that a sensitised guinea-pig passes rapidly into a condition of anti-anaphylaxis by injecting a very small dose of serum (·05 c.c.) subcutaneously into the peritoneum or into a vein. A fatal dose, or even a doubly fatal dose, can then be injected without any injurious effect. The immunity is produced within three or four hours after subcutaneous injection of the small dose and almost instantaneously after intravenous injection. This rapidity of action makes it possible, therefore, to render a patient immune to even minor doses of serum by a quick succession of injections. The method proposed consists in making three or four injections in rapid succession within a few minutes of each other, the dose of serum being augmented each time. In this way a condition of complete anti-anaphylaxis is produced. Guinea-pigs vaccinated in this way are found to be proof against any further injections, whether made into the peritoneun, spinal cord, or cerebrum.

### Origin of the Mononuclear Leucocytes.

At a recent meeting of the Berlin Medical Society Dr. Patella, of Sienna, read an interesting paper, in which he seeks to show that the large mononuclear leucocytes of the blood originate in the endothelial cells of the blood-vessels, pleura, peritoneum, and other vascular or lymphatic spaces. The increase in the number of these leucocytes in the blood-stream in cases of infectious fevers such as typhoid is accounted for by the general endothelial desquamation which takes place in such conditions, and it is just this desquamation which prepares the ground for such serious complications as endocarditis. From this point of view, therefore, the number of these mononuclear leucocytes is of considerable prognostic importance. But a similar mononucleosis may occur in old people owing to processes of arteriosclerosis, or may result from massage of the muscles, facts, which, as the author maintains, further confirm this view of the origin of the mononuclears from endothelial desquamation. The author is further of opinion that the lymphocytes of convalescence after an infectious fever are the same mononuclear leucocytes which have undergone changes during their free existence in the blood-stream. The presence of these lymphocytes and the return of the large mononuclear cells to the normal quantity are, therefore, favourable prognostic signs. In the subsequent discussion the views of the author were favourably criticised in regard to the endothelial origin of the large mononuclear cells, but their metamorphosis to lymphocytes was not credited, a separate origin for these latter from the lymphatics being considered more likely.

### Blood-pressure and the Pulse.

In a Manchester thesis, published in the *Medical Chronicle*, Dr. D. E. Core seeks to disentangle the complicated bearings of hæmomanometric observations upon clinical medicine. He arrives at the following deductions: The comparison of the daily variations of the blood-pressure and pulse, taken by the same observer under similar conditions, gives valuable information about the state of the patient and the course of his disease. The daily blood-pressure and pulse charts react very definitely to certain drugs, of which digitalis, sodium salicylate, potassium iodide, sodium nitrite, and ammonium carbonate are the most important. The daily variations of the blood-pressure and pulse show different relations to the daily excretion of urine, according as the case is one of heart failure secondary to renal disease, with arteriosclerosis, or heart failure due to endocarditis, without arteriosclerosis. The absolute value of the blood-pressure is of secondary importance compared with its daily variations. Patients suffering from diseases of the same organs show complete independence as regards the absolute value of their blood-pressure, whether this is considered from the standpoint of their age, sex, type of disease, or clinical course. The study of a daily blood-pressure chart, apart from the daily pulse chart, gives little or no information that is of any value. The most interesting part of this thesis is that which deals with the actions of certain drugs upon the heart, and the interpretations of the variations of pulse rate and blood-pressure which follow.

### The Prevention of Liver Abscess.

Amœbic abscess of the liver is a preventable disease in the vast majority of patients coming early under skilled medical treatment, and ought to become a very exceptional occurrence. Such is the belief of Major Leonard Rogers, and he explains in the *Journal of the Royal Army Medical Corps* how to ward off this formidable and fatal complication of amœbic dysentery. Preceding the stage of abscess formation is a more or less prolonged period of hepatitis with pyrexia, which, if acute, especially when preceded by dysentery, is not difficult to diagnose; it is in this stage of hepatitis that treatment is almost certain to be effectual, though the author also believes that even in some cases wherein actual suppuration had occurred the remedy has succeeded. In the insidious cases without localising signs a warning signal is sometimes given by the presence of a smart leucocytosis with a normal percentage of polynuclears. The basis of Major Rogers' prophylaxis is ipecacuanha, which must be given in large doses to obtain the therapeutic effect. To overcome the nausea which is so great an objection to the employment of this drug, doses of five grains put up in keratinised capsules are recommended, four to six being given at a time; another good plan is to coat freshly made pills with melted salol. Only one dose is given in the twenty-four hours, preferably late at night, on an empty stomach. The first sign of improvement is decrease of the hepatic pain, which is usually much lessened after one or two doses. In two to six days the temperature commonly falls to normal, and the acute symptoms subside. The enlargement of the liver disappears next, but it is advisable to continue the treatment in diminishing doses for at least two weeks, and preferably longer.

### The Virtues of Fresh Pineapple Juice.

According to the *Medical Record*, Dr. B. G. R. Williams, of Paris, Illinois, believes that the juice of the pineapple has digestive properties with reference to proteins, and is active in either acid or alkaline media. An enzyme called bromelin, destroyable by heat, is the agent of this activity, but citric acid is also present. He has never been able to obtain this enzyme in a preserved form, the digestive power always disappearing. It would seem to act as a destroyer of necrotic tissue in cases of quinsy, suppurative tonsilitis, and pharyngitis, and allows of easy evacuation of pus. It has the same effect on boils. The author has used it in a case of bedsores with good results. While not of much use in most gastric disorders, it has beneficial effects in hypochlorhydria and achylia. Its great advantage over other enzymes is its pleasant taste, it being only necessary to eat a piece of fresh fruit. The author remarks on its value as a preventive when about to eat a "Thanksgiving dinner" or other meal which generally causes indigestion.

### Babinski's Sign in Diphtheria.

Dr. J. D. Rolleston, in an article reprinted from the *Review of Neurology and Psychiatry*, discusses the question of the presence of Babinski's sign in diphtheria. His paper deals with a series of 877 cases of the disease in which the plantar reflex was investigated in the course of the last four years. In 172 or 19.6 per cent. of cases an extensor response of the great toe, with extension or flexion of the other toes, was found to be present for varying periods. In 29 flexion alternated with extension, and in 676 the normal flexor response was obtained. In no case was there absence of any response. In no case again was strychnine administered, as the occurrence of the sign after large doses of this drug has been noted. From the author's analysis of his cases he comes to the following conclusions: (1) Babinski's sign is found in a considerable percentage of all cases of diphtheria, the character of the response being rapid, deliberate, or intermediate in character; (2) the extensor response in this disease is not confined to infants, but may be obtained, though with decreasing frequency and duration, especially after the eighth year, until adult life; (3) it is essentially a phenomenon of the acute stage, in most cases being replaced by flexion in convalescence. Transition stages often exist in which various forms of response may be obtained; (4) the sign is not pathognomonic of diphtheria, since it occurs in other acute infections such as scarlet fever, typhoid, lobar pneumonia, etc., but it has a certain diagnostic value, since it is met with more frequently in diphtheria than in non-diphtheritic angina; (5) it is more frequent and persistent in severe than in mild attacks of the disease, and its presence has therefore a certain prognostic value; (6) it is not associated with any special condition of the tendon jerks, and is never accompanied by ankle-clonus; and (7) it is probably due to a transitory perturbation of the pyramidal system by the circulating toxins, comparable to the slight degree of meningeal reaction which is a frequent occurrence in acute infections.

### Morphia in Gastric Crises of Tabes.

The patient who is the subject of Dr. P. A. Ostankow's contribution to the *Neurologisches Centralblatt* had always been free from gastric crises until his first morphia injection. After using the drug regularly for two years, the crises occurred at regular intervals, accompanied by an elevation of temperature and a marked fall in the amount of urine passed. From the day that the use of morphine was discontinued entirely the crises disappeared. The author has found similar symptoms in cases of intoxication by heroin. It would therefore seem that morphia and the other derivatives of opium can, by continual use, provoke true gastric crises in tabetics. It is true that morphia, in common with other anti-neuralgic remedies, such as antipyrin, phenacetin, aspirin, etc., keep off attacks of pain and crises for a short time, but it would seem equally true that the continued use of any of these remedies may in some cases bring about the more frequent return of the symptoms. It is therefore wise to administer them very sparingly.

### Cancer in Sierra Leone.

Under the heading, "The Spread of Cancer among the descendants of the liberated Africans or Creoles of Sierra Leone," Dr. W. Renner of that colony contributes a thoughtful paper to the *Journal of Tropical Medicine and Hygiene*. In this he shows that the aboriginal native was, and still is, remarkably free from cancerous growths, and this after making due allowance for cases which have escaped the notice of the medical officers, or have remained under the treatment of the witch-doctors. A remarkable contrast is, however, afforded by the descendants of liberated Africans or Creoles. These, being derived from tribes scattered all over the African Continent, possess no tribal customs in common, and, unlike the aborigines, have adopted the manners and customs of their liberators. Being for the most part well-to-do, they are flesh-eaters, whereas the indigenous race exist largely on grain and vegetables. The author is not convinced, as yet, that the theory which makes flesh-eating responsible for the dissemination of cancer is true, but he points out that the steady increase during the past few decades of cancer among meat-eating Creoles, and the absence of such an increase in the vegetarian aborigines, would seem to furnish proof of the truth of that theory. He finds himself unable to endorse the opinion of Dr. Hearsey, principal medical officer of British Central Africa, that the suppression of suckling among European women has a direct relation to the greater incidence of carcinoma mammæ among them as compared with native women, who always suckle their young, and for a lengthy period. The Creoles are excellent mothers, and suckle their young for as long a time as the native women, and yet carcinoma mammæ is on the increase among the former and not among the latter. Heredity cannot bear the blame, for the Creole community is derived from many sources, and statistical inquiry does not support the theory of its being a causative factor. The author can only account for the increase of cancer among the Creoles by supposing that a hankering after the methods of a so-called civilisation has had a disastrous result on the physical fibre of this community, an opinion in which he is indirectly supported by the fact that he has discovered other traces of deterioration in it; for example, a marked increase in the incidence of dental caries among Creole children, from which the native race continues, as always, to be practically exempt. He would welcome research into the question and the collection of data, in order, if possible, to find a remedy for an evil which is increasing.

### Syphilis of the Ear.

A Summary of the ways in which syphilis may manifest its presence at its various stages in the ear is given by G. Berruyer in *Le Bulletin Médical*. Syphilitic papules and gummata may attack the external ear in the same way as any other skin-surface, but the most serious manifestations of the disease are, as might be expected, found in connection with the internal ear. In hereditary syphilis of the new-born, suppuration of the middle ear accompanies the catarrhal conditions which exist in the naso-pharynx. It arises insidiously, without pain or general or local reaction, and, if bilateral, may result in deaf-mutism. At a later age specific ulcerations of the naso-pharynx may be accompanied by otitis media, deafness, and retracted drum. From twenty to thirty years of age a dry otitis occurs, leading to deafness, and the patient may exhibit signs of the taint in the eyes and teeth. In the acquired variety of the disease, mucous patches may lead to inflammation of the eustachian tube, and end in disease of that organ and the tympanum. Diagnosis rests on the resistance of the condition to all except anti-syphilitic remedies, and on the diminished bone conductivity, which shows that the internal ear is implicated. Tertiary syphilis in the naso-pharynx may cause similar conditions in the internal ear. Deafness, occurring suddenly about the time of puberty without any apparent cause, may be a tertiary manifestation. Deafness is absolute, and the outlook very bad. Again, the deafness may develop slowly in some cases. In acquired syphilis deafness is brought about by the obliteration of the labyrinthine arteries. Neuralgia, becoming worse at night, is a symptom. Deafness is sudden, quickly absolute, and is beyond hope of cure.

